# Fascicles and the interfascicular matrix show decreased fatigue life with ageing in energy storing tendons^☆^

**DOI:** 10.1016/j.actbio.2017.03.024

**Published:** 2017-07-01

**Authors:** Chavaunne T. Thorpe, Graham P. Riley, Helen L. Birch, Peter D. Clegg, Hazel R.C. Screen

**Affiliations:** a Institute of Bioengineering, School of Engineering and Materials Science, Queen Mary University of London, Mile End Road, London E1 4NS, UK; b School of Biological Sciences, University of East Anglia, Norwich Research Park, Norwich NR4 7TJ, UK; c Institute of Orthopaedics and Musculoskeletal Science, University College London, Royal National Orthopaedic Hospital, Stanmore HA7 4LP, UK; d Department of Musculoskeletal Biology, Institute of Ageing and Chronic Disease, University of Liverpool, Leahurst Campus, Neston CH64 7TE, UK

**Keywords:** Tendon, Fascicle, Interfascicular matrix, Mechanical testing, Fatigue resistance, Creep

## Abstract

Tendon is composed of rope-like fascicles bound together by interfascicular matrix (IFM). The IFM is critical for the function of energy storing tendons, facilitating sliding between fascicles to allow these tendons to cyclically stretch and recoil. This capacity is required to a lesser degree in positional tendons. We have previously demonstrated that both fascicles and IFM in energy storing tendons have superior fatigue resistance compared with positional tendons, but the effect of ageing on the fatigue properties of these different tendon subunits has not been determined. Energy storing tendons become more injury-prone with ageing, indicating reduced fatigue resistance, hence we tested the hypothesis that the decline in fatigue life with ageing in energy storing tendons would be more pronounced in the IFM than in fascicles. We further hypothesised that tendon subunit fatigue resistance would not alter with ageing in positional tendons. Fascicles and IFM from young and old energy storing and positional tendons were subjected to cyclic fatigue testing until failure, and mechanical properties were calculated. The results show that both IFM and fascicles from the SDFT exhibit a similar magnitude of reduced fatigue life with ageing. By contrast, the fatigue life of positional tendon subunits was unaffected by ageing. The age-related decline in fatigue life of tendon subunits in energy storing tendons is likely to contribute to the increased risk of injury in aged tendons. Full understanding of the mechanisms resulting in this reduced fatigue life will aid in the development of treatments and interventions to prevent age-related tendinopathy.

**Statement of Significance:**

Understanding the effect of ageing on tendon-structure function relationships is crucial for the development of effective preventative measures and treatments for age-related tendon injury. In this study, we demonstrate for the first time that the fatigue resistance of the interfascicular matrix decreases with ageing in energy storing tendons. This is likely to contribute to the increased risk of injury in aged tendons. Full understanding of the mechanisms that result in this reduced fatigue resistance will aid in the development of treatments and interventions to prevent age-related tendinopathy.

## Graphical abstract

**Figure:**
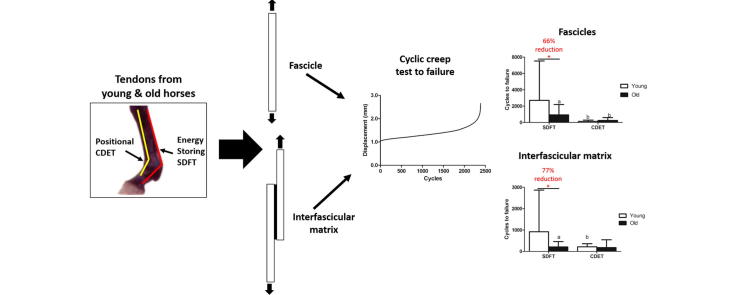

## 1 Introduction

Tendons attach muscle to bone and transfer force generated by muscle contraction to the skeleton, facilitating movement. The ability to withstand large unidirectional forces is provided by their structure; tendons are hierarchical fibre-composite materials, in which type I collagen molecules group together to form subunits of increasing diameter, the largest of which is the fascicle [1]. Adjacent fascicles are bound together by a looser matrix, termed the interfascicular matrix (IFM; sometimes referred to as the endotenon).

Tendons can broadly be divided into two categories depending on their function, those that act purely to position the limb and those that act as elastic springs during exercise, storing energy and thus reducing the energetic cost of locomotion [2], [3]. Energy-storing tendons, such as the human Achilles tendon and equine superficial digital flexor tendon (SDFT), are subjected to high forces and are more compliant than positional tendons, such as the human anterior tibialis tendon and equine common digital extensor tendon (CDET), to allow the elongation required for maximal energy storage and return [4], [5], [6]. The large extensions required by energy storing tendons are facilitated by sliding between fascicles, allowing the tendon to stretch further than its constituent fascicles [5]. This sliding behaviour is governed by the IFM [5]. Both the IFM and fascicles from energy storing tendons exhibit superior elasticity and fatigue resistance when compared to those from positional tendons [7], [8]. The specialised properties of the subunits in energy storing tendons likely provide the whole tendon with improved fatigue resistance so that it can resist the large, repetitive stresses and strains it experiences during use.

Despite these specialisations, energy-storing tendons are particularly prone to injury [9], [10], which is thought to occur as a result of accumulation of microdamage within the tendon matrix rather than acute injury [11]. The incidence of injury increases with ageing, both within the human Achilles [10], [12] and equine SDFT [13], [14], indicating a reduction in tendon fatigue resistance. We have previously demonstrated that IFM stiffness increases in the aged energy storing SDFT, decreasing the capacity for fascicle sliding [7], [15]. Further, fascicle fatigue resistance decreases with ageing specifically in energy storing tendons [16]. Both these age-related alterations are likely to contribute to the increased risk of injury with ageing. However, the effect of ageing on the fatigue resistance of the IFM is yet to be established. As our previous studies have highlighted the important contribution of the IFM to the healthy function of energy storing tendons [5], [7], we therefore tested the hypothesis that the decline in fatigue life with ageing in the energy storing SDFT would be more pronounced in the IFM than in fascicles. We further hypothesised that both fascicle and IFM fatigue life would not alter with ageing in the positional CDET.

## 2 Materials and methods

### 2.1 Sample collection and preparation

Distal forelimbs were collected from horses aged 3 to 7 years (n = 4; young age group) and 17 to 20 years (n = 4; old age group) euthanased at a commercial equine abattoir. The Animal (Scientific Procedures) Act 1986, Schedule 2, does not define collection from these sources as a scientific procedure. The forelimb SDFT and CDET were removed from the limbs within 24 h of death, and wrapped in tissue paper moistened with phosphate buffered saline (PBS) followed by tinfoil to prevent sample dessication, before freezing at −80 °C. While it was not possible to obtain a detailed history for the horses, none of the tendons had clinical or macroscopic evidence of injury. Prior to testing, tendons were thawed and both fascicles, approximately 30 mm in length, and groups of two fascicles, bound together by IFM were isolated from the mid-metacarpal region of the tendon as described previously (6–8 per tendon (total = 24–32 samples per condition)) [5], [17]. Fascicles were maintained on tissue paper moistened with Dulbecco’s modified eagle medium (DMEM) to main hydration during testing.

### 2.2 Determination of fascicle fatigue properties

Fascicle diameter was determined using a laser micrometer as described previously, using the smallest diameter to calculate cross-sectional area, assuming a circular cross-section [5]. Fascicles were clamped in custom-made loading chambers [18], with a clamp-to-clamp distance of 10 mm. The fatigue properties of the fascicles were measured using a mechanical test machine, equipped with a 22 N load cell (Electroforce 5500, TA instruments, Delaware, USA), located in a cell culture incubator (37 °C, 20% O_2_, 5% CO_2_). To remove any slack within the samples, a pre-load of 0.1 N was applied prior to the start of the test. We have previously established that fascicle failure strain is more consistent between samples than failure stress [5]. Accordingly, one loading cycle to a displacement of 1 mm (10% strain, equivalent to 50% of predicted failure strain [17]) was applied to establish an appropriate and consistent peak load, which was subsequently applied to the fascicles in a cyclic manner at a frequency of 1 Hz until sample failure. The minimum load applied in each cycle was 0.1 N. Load and displacement data were recorded continuously throughout the test (frequency: 100 Hz).

### 2.3 Determination of IFM fatigue properties

Samples were prepared for IFM fatigue testing as described previously [5], [15]. Briefly, transverse cuts were made in the opposing ends of 2 fascicles bound together by IFM, to leave a 10 mm length of IFM for testing in shear. The intact end of each fascicle was secured in the loading chambers and IFM fatigue properties were determined as described for the fascicle tests. A pre-load of 0.02 N was applied to remove any sample slack. IFM failure extension is more consistent between cycles than failure force [5], therefore one loading cycle of 1 mm displacement was applied, (equivalent to 50% of the predicted failure extension) [5], to find the peak load. This load was subsequently applied to the IFM cyclically at a frequency of 1 Hz until sample failure. The minimum load applied in each cycle was 0.02 N. Displacement and load data were recorded throughout the test (frequency: 100 Hz).

### 2.4 Data analysis

For each test, the number of cycles to failure was recorded. Creep curves to failure were plotted using the minimum and maximum displacement data. The gradient of the secondary portion of the resultant creep curves was calculated.

Force extension curves were plotted from the load and displacement data. Hysteresis over cycles 1–10, 11–20, the middle 10 cycles and the last 10 cycles prior to failure was calculated as described previously [8].

Fascicle laxity (defined as the minimum displacement at a particular cycle number) and elongation (defined as the maximum displacement at a particular cycle number) were calculated for the 1st and 10th cycles, and the cycle prior to failure. It was not possible to calculate IFM laxity or elongation for cycle 1, as the low forces applied in this load controlled experiment required several cycles to fully stabilise, therefore laxity and elongation at cycle 10 and the cycle prior to failure were calculated. A comparison of the fascicle and IFM data from the young SDFT and CDET has been published previously [8].

### 2.5 Statistical analysis

Data were averaged from all tests and are displayed as mean ± SD. Statistical differences between age groups and tendon types were determined by fitting a general linear model to the data, including donor, tendon type, and horse age included as factors (Minitab 17). Inclusion of donor (individual horse) as a factor takes into account each replicate measure/fascicle whilst allowing for clustering around a donor, ensuring that fascicles from the same tendon are not considered as independent biological replicates. Data were tested for normality (Anderson–Darling test) and those that did not follow a normal distribution were transformed using a Box-Cox transformation. Post-hoc comparisons were performed using Tukey’s test. To determine if the reduction in cycles to failure was significantly different between fascicles and IFM, linear regression analysis was performed.

To assess correlations between initial mechanical parameters (hysteresis and elongation over cycles 1 to 10) and the number of cycles to failure, Spearman correlation coefficients were calculated for aged fascicles (correlations for young fascicles have been reported previously) [8]. It was not possible to calculate IFM parameters relative to the first cycle, therefore correlations were not calculated for the IFM.

## 3 Results

### 3.1 Effect of ageing on fascicle fatigue properties

**Fig. 1:**
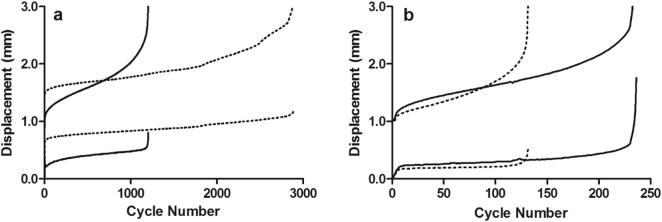
Typical creep curves for fascicles from young (^……….^) and old () SDFTs (a) and CDETs (b). The minimum and maximum displacement reached in each cycle is plotted against cycle number. Note the difference in x-axis scale between graphs.

**Fig. 2:**
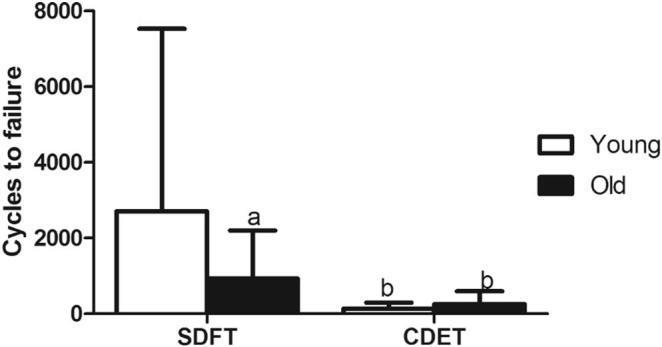
Mean number of cycles to failure for fascicles from the young and old SDFT and CDET. Data are displayed as mean ± SD. ‘a’ indicates significant differences between age groups (p ≤ 0.05), ‘b’ indicates significant differences between tendon types (p ≤ 0.05).

Fatigue properties for fascicles from the SDFT and CDET are shown in Table S1. Typical maximum and minimum creep curves for fascicles are shown in Fig. 1. Fascicle fatigue resistance was significantly greater in the SDFT than in the CDET, both in young and old fascicles (p ≤ 0.003). The number of cycles to failure decreased significantly, by 65.7% with ageing in fascicles from the SDFT (p = 0.05), but was not altered with age in those from the CDET (Fig. 2).

**Fig. 3:**
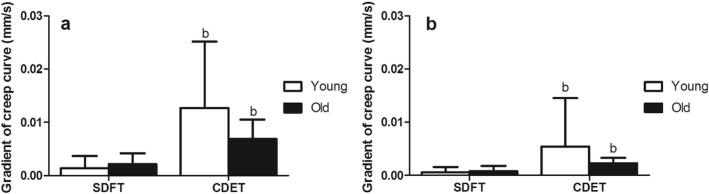
Gradient of the maximum (a) and minimum (b) creep curves of young and old fascicles from the SDFT and CDET. Data are displayed as mean ± SD. ‘b’ indicates significant differences between tendon types (p < 0.05).

There was a trend towards an increase in the gradient of the maximum creep curve with ageing in the SDFT, but this was not significant (p = 0.1; Fig. 3). The gradients of the maximum and minimum creep curves did not alter with ageing in the CDET. Maximum and minimum creep curve gradients were significantly greater in the CDET than in the SDFT in both age groups (Fig. 3).

**Fig. 4:**
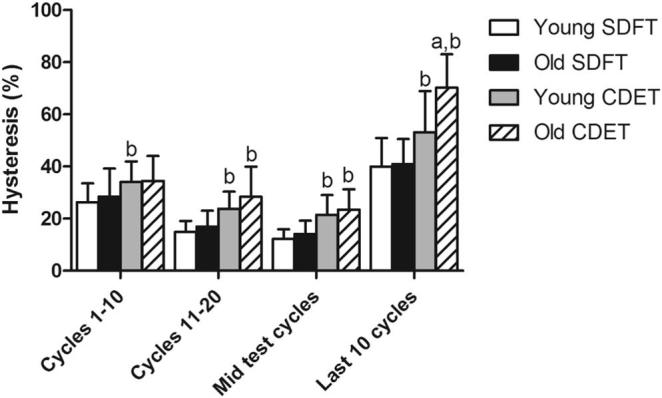
Hysteresis at different points throughout fatigue testing in young and old fascicles from the SDFT and CDET. Data are displayed as mean ± SD. ‘a’ indicates significant differences between age groups (p ≤ 0.05), ‘b’ indicates significant differences between tendon types (p ≤ 0.05).

In aged fascicles, hysteresis over the duration of the test followed a similar trend to that seen previously in young fascicles [8], decreasing until the mid-test cycles, and then increasing significantly in the final 10 cycles prior to failure (p < 0.001). Ageing did not cause any alterations in hysteresis in fascicles from the SDFT, however hysteresis increased significantly with ageing in CDET fascicles in the 10 loading cycles prior to failure (Fig. 4). Hysteresis throughout the test cycles was significantly greater in the CDET than in the SDFT, both in young and old fascicles.

**Fig. 5:**
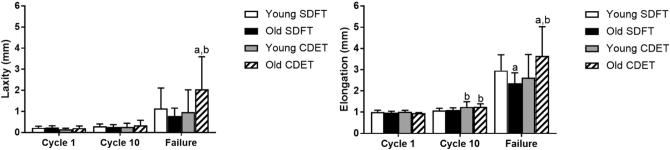
Fascicle laxity (a) and elongation (b) in the SDFT and CDET from young and old horses. Data are displayed as mean ± SD. ‘a’ indicates significant differences between age groups (p < 0.05), ‘b’ indicates significant differences between tendon types (p < 0.05).

Fascicle laxity by cycle 10 did not differ with ageing or between tendon types (Fig.5a). Fascicle laxity increased significantly with ageing in the CDET in the cycle prior to failure (p < 0.001; Fig.5a). Fascicle elongation at cycle 10 was significantly greater in the CDET than in the SDFT in both age groups (p ≤ 0.005; Fig.5b). At the cycle prior to failure, fascicle elongation decreased with ageing in the SDFT, but increased with ageing in the CDET (p < 0.03).

When considering the relationships between initial mechanical parameters and cycles to failure in aged fascicles, initial hysteresis was positively correlated with elongation at cycle 10 in both tendon types (Table 1), similar to the response seen in young fascicles that we have reported previously [8]. The number of cycles to failure showed a negative correlation with elongation in both tendon types, and with hysteresis in the SDFT only (Table 1).

**Table 1:** Correlations between initial mechanical testing parameters (hysteresis and elongation at cycle 10) and the number of cycles to failure in fascicles from the old SDFT and CDET. NS = not significant.

	Elongation (mm)		Cycles to failure	
	SDFT	CDET	SDFT	CDET
Hysteresis (%)	p = 0.014 r = 0.63	p = 0.012 r = 0.80	p = 0.013 r = −0.60	NS
Elongation (mm)	–	–	p = 0.034 r = −0.54	p = 0.032 r = −0.73

### 3.2 Effect of ageing on IFM fatigue properties

**Fig. 6:**
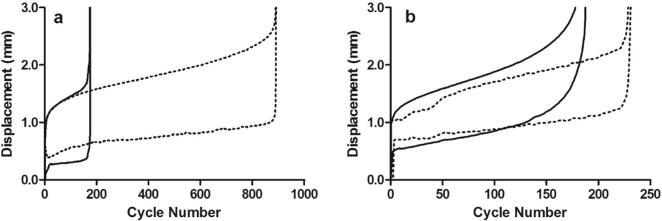
Typical creep curves for the IFM from young (^……….^) and old () SDFTs (a) and CDETs (b). The maximum displacement reached in each cycle is plotted against cycle number. Note the difference in x-axis scale between graphs.

**Fig. 7:**
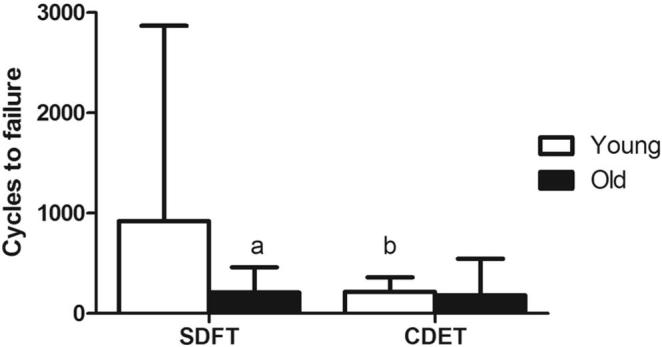
Mean number of cycles to failure in the IFM from young and old SDFT and CDET. Data are displayed as mean ± SD. ‘a’ indicates significant differences between age groups (p < 0.05), ‘b’ indicates significant differences between tendon types (p < 0.05).

**Fig. 8:**
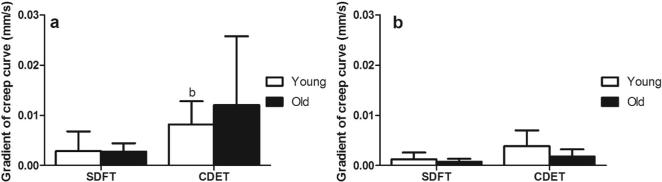
Gradient of the maximum (a) and minimum (b) creep curves of young and old IFM from the SDFT and CDET. Data are displayed as mean ± SD. ‘b’ indicates significant differences between tendon types (p < 0.05).

**Fig. 9:**
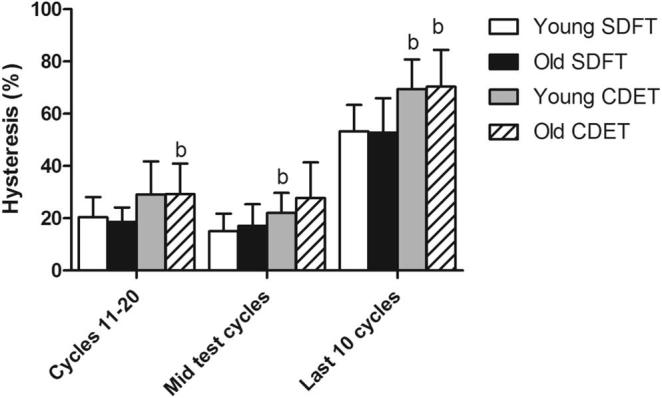
Hysteresis at different points throughout fatigue testing in young and old IFM from the SDFT and CDET. Data are displayed as mean ± SD. ‘b’ indicates significant differences between tendon types (p < 0.05).

Fatigue properties of the IFM in the SDFT and CDET are shown in Table S2. Typical maximum and minimum creep curves for the IFM are shown in Fig. 6. The number of cycles to failure decreased significantly with ageing in the SDFT IFM (p = 0.03), with an overall decrease in fatigue resistance of 77.4%. The degree of reduction in fatigue resistance was not significantly different between fascicles and IFM in the SDFT. Number of cycles to failure was not altered with age in the CDET IFM (Fig. 7). In aged tendons, there was no longer any significant difference in the number of cycles to failure between tendon types (Fig. 7). The gradient of the maximum and minimum creep curves was not altered with ageing in either tendon type (Fig. 8). Ageing did not cause any alterations in IFM hysteresis in either tendon type (Fig. 9). Hysteresis was consistently greater in the CDET than in the SDFT in both age groups.

**Fig. 10:**
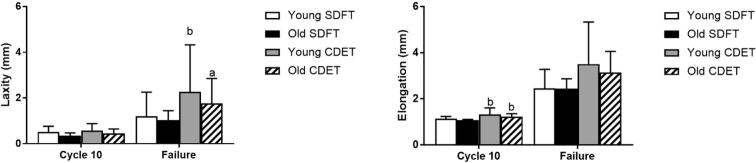
IFM laxity (a) and elongation (b) at cycle 10 and the cycle prior to failure, in the SDFT and CDET from young and old horses. Data are displayed as mean ± SD. ‘a’ indicates significant differences between age groups (p < 0.05), ‘b’ indicates significant differences between tendon types (p < 0.05).

IFM laxity did not alter with ageing or tendon type at cycle 10, but was significantly greater in the young CDET than in the young SDFT at the cycle prior to failure (p < 0.001; Fig.10a)). This difference was lost with ageing, due to a decrease in CDET IFM laxity (p < 0.001; Fig.10a)). IFM elongation was significantly greater in the CDET than in the SDFT in both age groups (p ≤ 0.01; Fig.10b) at cycle 10, and was unaffected by ageing. There were no alterations in IFM elongation with ageing or between tendon types at the cycle prior to failure.

## 4 Discussion

This is the first study to investigate age-related alterations in the fatigue behaviour of the tendon IFM, and also provide a comprehensive analysis of age-related alterations in fascicle fatigue resistance. The results support the hypothesis, demonstrating an age-related decline in fatigue life of subunits from energy storing tendons. However, there was no significant difference in the degree of reduction in fatigue life between the fascicles and IFM of the energy storing SDFT. In further support of the hypothesis, both fascicle and IFM fatigue resistance remained unchanged with ageing in the positional CDET.

The limitations associated with IFM and fascicle fatigue testing using the experimental set up in this study, including possible sample damage prior to testing, the unbalanced shear design used for IFM testing, and inability to calculate IFM mechanical properties during the first loading cycle have been discussed previously [8].

Though several studies have investigated alterations in tendon mechanical properties as a function of ageing [15], [19], [20], [21], few have assessed the effect of fatigue loading on aged tendon, either at the level of the whole tendon, or within tendon sub-units. A study by Kietrys et al. [22], using an *in vivo* rat overuse model has shown that repetitive loading in aged individuals resulted in greater tendon inflammation and reduced limb agility compared with young tendons that had undergone the same loading regime [22]. It has also been shown that, while there is no age-related difference in the amount of elongation of the energy storing human patellar tendon that occurs due to cyclic loading *in vivo;* this elongation takes longer to recover in aged individuals [23]. Similar results were obtained when viable rat tail tendon fascicles were cyclically loaded *in vitro* and then allowed to recover [23]. Taken together, these results support those presented in the current study, indicating the presence of age-related alterations to tendon structure which decrease their ability to withstand repetitive loading.

When considering the response of the IFM to fatigue loading, we have previously demonstrated superior fatigue resistance of the IFM in the energy storing SDFT when compared to the CDET [8]. In the current study, fatigue life of the SDFT IFM decreased with ageing. While the percentage decrease in number of cycles to failure was greater in the IFM than in the fascicles, this difference was not significant. However, the number to cycles to failure was highly variable, suggesting that any difference in the reduction of fatigue life with ageing may have been missed due to noise in the data. It is interesting to note that, while fascicles from the aged SDFT still exhibited greater fatigue life than those from the CDET, there were no longer any apparent differences in IFM fatigue life between the aged SDFT and CDET. Looking more closely at the fatigue failure of the SDFT IFM, it was notable that while the number of cycles to failure was significantly decreased with ageing in the energy storing SDFT, we did not identify any alterations in the creep response or energy loss during each loading cycle prior to failure, indicating that the viscoelastic properties of the IFM do not decline with ageing. Indeed, creep curves for the IFM remain remarkably similar in the SDFT between age groups (Fig. 6), but the aged IFM fails after far fewer cycles, indicating that the earlier failure of the IFM in the aged SDFT may be a result of localised areas of stiffening within the IFM, caused by improper repair of microdamage, which reduces the mechanical competence of the tissue.

Supporting this, we have previously shown that the rate of protein turnover is decreased in the aged IFM, suggesting a reduced ability to repair microdamage within this region [24]. In addition, we have also identified changes in the mechanical response of the IFM to quasi-static loading, demonstrating that the initial elongated toe response seen in the SDFT IFM is lost with ageing, reducing the capacity for interfascicular sliding [5], [7]. The mechanisms governing IFM sliding behaviour are yet to be fully determined, however we have previously identified the presence of lubricin and elastin within the IFM, with lubricin likely facilitating sliding between fascicles and elastin governing recoil [25]. It is possible that age-related alterations occur to these proteins, and these structural changes result in the reduced fatigue resistance seen with ageing. This remains an important area for future research.

Fascicles showed a response to ageing similar to that seen in the IFM, with a decrease in fatigue properties in the SDFT only. However, unlike the response observed in the IFM, aged SDFT fascicles were still able to resist significantly more cycles to failure than their counterparts from the CDET. While CDET fascicle fatigue resistance did not alter with ageing, fascicle elongation and laxity increased with age in the cycle prior to failure; this is likely related to the age-related increase in fascicle failure strain previously identified [7]. We also observed an increase in fascicle diameter with ageing in the CDET. As the peak load measured did not increase concomitantly, applied stresses were significantly lower with increasing age in the CDET. The increase in fascicle diameter in the aged CDET may be due to increased spacing within the fascicles rather than any alterations in fascicle composition; assessing age-related changes in intra-fascicular spacing remains an important area for future research. It is possible that the reduced peak stress applied to the CDET may have resulted in over-estimation of CDET fascicle fatigue properties in aged individuals. Indeed, the average number of cycles to failure was slightly higher in aged CDET fascicles, although this was not significant.

It is possible that the decreased fatigue life of SDFT fascicles with ageing is due to alterations in fascicle substructure with ageing. Our previous work has demonstrated two independent age-related mechanisms of fatigue failure in fascicles from the energy storing SDFT. Fascicles from young tendons have a helical substructure which allows efficient extension and recoil [26]. Fatigue loading results in alterations to the helix substructure, reducing the ability of energy-storing tendons to recoil and recover from loading [17]. In SDFT fascicles from aged horses, the helix structure is already compromised [16], such that fatigue loading results in increased sliding between the collagen fibres within the fascicles, and more extensive damage within the matrix [16]. Considering these results in light of our current findings, it is possible that, in young SDFT fascicles cyclically loaded to failure, loading is first managed by extension and recoil of the helix. After a certain number of cycles, this is lost, and fibre sliding likely occurs. By contrast, in old SDFT fascicles, the compromised helix may result in the decreased fatigue life observed in the current study. It is interesting to note that young SDFT fascicles are able to elongate slightly further before failure than those from aged tendons; while a small proportion of this elongation may be conferred by the unwinding of the helix, this cannot account fully for the difference in elongation with ageing in SDFT fascicles, such that there may be additional, as yet unidentified, ageing changes within the SDFT.

## 5 Conclusion

We observed an age-related decline in fatigue life of subunits from energy storing tendons. By contrast, fatigue resistance of the subunits of positional tendons were unaffected by ageing. These findings indicate that IFM and fascicle fatigue life are equally important for the fatigue resistance of the whole tendon, and the age-related decline in the fatigue life of tendon subunits is likely to contribute to the increased risk of injury, and likely reduced fatigue resistance, in aged tendons. Full understanding of the mechanisms resulting in this reduced fatigue life will aid in the development of treatments and interventions to prevent age-related tendinopathy.
