# Supplementary material for: Fascicles and the interfascicular matrix show decreased fatigue life with ageing in energy storing tendons
Source: Acta Biomater. 2017 Jul 1;56:58–64. doi: 10.1016/j.actbio.2017.03.024 (PMC5486374; doi:10.1016/j.actbio.2017.03.024)
Supplement: Supplementary data [file mmc1.docx]

**Supplementary Information**

|  | **SDFT** | | **CDET** | |
| --- | --- | --- | --- | --- |
|  | **Young** | **Old** | **Young** | **Old** |
| **Diameter (mm)** | 0.33 ± 0.14 | 0.31 ± 0.06 | 0.37 ± 0.09*^e^* | 0.45 ± 0.09*^b,f^* |
| **Load applied (N)** | 3.68 ± 1.46 | 3.76 ± 1.96 | 4.80 ± 1.88 | 4.20 ± 2.26 |
| **Stress applied (MPa)** | 52.81 ± 28.62 | 45.89 ± 13.76 | 47.99 ± 22.26 | 25.20 ± 8.45*^c,f^* |
| **Number of cycles to failure** | 2709 ± 4819 | 929 ± 1271*^a^* | 139 ± 157*^f^* | 254 ± 339*^e^* |
| **Gradient of maximum creep curve** | 0.0014 ± 0.0023 | 0.0021 ± 0.0021 | 0.013 ± 0.012*^f^* | 0.069 ± 0.036 *^f^* |
| **Gradient of minimum creep curve** | 0.00059 ± 0.00099 | 0.00081 ±0.00097 | 0.0054 ± 0.0091*^f^* | 0.0023 ±  0.0010 *^f^* |
| **Hysteresis (%): Cycle 1-10**  **Cycle 11-19**  **Mid test cycles**  **Last 10 cycles** | 26.26 ± 7.31  14.91 ± 4.23  12.22 ± 3.70  39.93 ± 11.05 | 28.44 ± 10.77  16.99 ± 6.04  14.15 ± 5.09  40.96 ± 9.55 | 34.05 ± 7.92*^e^*  23.77 ± 6.60*^f^*  21.48 ± 7.56*^f^*  53.12 ± 15.86*^f^* | 34.42 ± 9.64  28.34 ± 11.59*^c^*  23.48 ± 7.79 *^c^*  70.19 ± 12.88*^c,f^* |
| **Laxity: Cycle 1**  **Cycle 10**  **Cycle prior to failure** | 0.22 ± 0.075  0.29 ± 0.11  1.14 ± 0.97 | 0.24 ± 0.079  0.26 ± 0.11  0.78 ± 0.37 | 0.15 ± 0.053  0.26 ± 0.18  0.96 ± 1.06 | 0.21 ± 0.10  0.34 ± 0.24  2.05 ± 1.55 *^c,f^* |
| **Elongation: Cycle 1**  **Cycle 10**  **Cycle prior to failure** | 1.01 ± 0.080  1.09 ± 0.098  2.96 ± 0.74 | 0.98 ± 0.066  1.10 ± 0.11  2.37 ± 0.49*^a^* | 1.02 ± 0.066  1.25 ± 0.25*^f^*  2.62 ± 1.10 | 0.96 ± 0.027  1.25 ± 0.15*^e^*  3.66 ± 1.37*^b,f^* |
|  |  |  |  |  |

Table S1. Fatigue properties of fascicles from the young and old SDFT and CDET. Data are displayed as mean ± SD. Significant differences between age groups identified by: *^a^* p ≤ 0.05; *^b^* p ≤ 0.01; *^c^* p ≤ 0.001. Significant differences between tendon types identified by: *^d^* p ≤ 0.05; *^e^* p ≤ 0.01; *^f^* p ≤ 0.001.

|  | **SDFT** | | **CDET** | |
| --- | --- | --- | --- | --- |
|  | **Young** | **Old** | **Young** | **Old** |
| **Load applied (N)** | 1.15 ± 0.85 | 1.67 ± 1.08 | 1.29 ± 1.28 | 1.79 ± 1.11 |
| **Number of cycles to failure** | 921 ± 1947 | 208 ± 253*^a^* | 215 ± 145*^e^* | 181 ± 364 |
| **Gradient of maximum creep curve** | 0.0029 ± 0.0039 | 0.0028 ± 0.0017 | 0.0082 ± 0.0046*^e^* | 0.012 ± 0.014 |
| **Gradient of minimum creep curve** | 0.0012 ± 0.0014 | 0.00077 ± 0.00058 | 0.0039 ± 0.0031 | 0.0018 ± 0.0014 |
| **Hysteresis (%): Cycle 11-20**  **Mid test cycles**  **Last 10 cycles** | 20.38 ± 7.73  15.08 ± 6.69  53.32 ± 10.06 | 18.54 ± 5.60  17.04 ± 8.30  52.83 ± 13.14 | 29.06 ± 12.74  22.06 ± 7.63*^e^*  69.44 ± 11.38*^e^* | 29.26 ± 11.69*^d^*  27.78 ± 13.62  70.34 ± 14.08*^f^* |
| **Laxity (mm):** **Cycle 10**  **Cycle prior to failure** | 0.52 ± 0.24  1.20 ± 1.05 | 0.35 ± 0.12  1.04 ± 0.41 | 0.57 ± 0.31  2.27 ± 2.05 *^f^* | 0.45 ± 0.20  1.77 ± 1.08 *^c^* |
| **Elongation (mm): Cycle 10**  **Cycle prior to failure** | 1.13 ± 0.11  2.45 ± 0.83 | 1.07 ± 0.03  2.44 ± 0.43 | 1.32 ± 0.29*^e^*  3.51 ± 1.83 | 1.22 ± 0.12*^d^*  3.14 ± 0.92 |

Table S2. Fatigue properties of IFM from the young and old SDFT and CDET. Data are displayed as mean ± SD. Significant differences between age groups identified by: *^a^* p ≤ 0.05; *^b^* p ≤ 0.01; *^c^* p ≤ 0.001. Significant differences between tendon types identified by: *^d^* p ≤ 0.05; *^e^* p ≤ 0.01; *^f^* p ≤ 0.001.
